# The severity of dry eye symptoms and risk factors among university students in Saudi Arabia: a cross-sectional study

**DOI:** 10.1038/s41598-024-65297-6

**Published:** 2024-07-02

**Authors:** Anas Alqurashi, Hatim Almaghrabi, Muath Alahmadi, Abdulaziz Alotaibi, Bandar Alotaibi, Abdulaziz Jastaniah, Ameera Bukhari, Mohammad Binhussein, Basant Othman, Amer Khojah

**Affiliations:** 1 College of Medicine, Umm Al-Qura University, Makkah, Saudi Arabia; 2 Biotechnology Department, College of Science, Taif University, Taif, Saudi Arabia; 3 Department of Ophthalmology, Alnoor Hospital, Makkah, Saudi Arabia; 4 Pediatric Department, College of Medicine, Umm Al-Qura University, Makkah, Saudi Arabia; 5 Medicine Department, College of Medicine, Umm Al-Qura University, Makkah, Saudi Arabia

**Keywords:** Dry eye syndrome, Dry eye disease, Computer vision syndrome, University students, Digital screen, Saudi Arabia, Ocular surface disease index, Monitor filters, Eye diseases, Lifestyle modification

## Abstract

Dry eye syndrome (DES) is a tear film disorder caused by increased tear evaporation or decreased production. The heavy workload on the eye and the increased usage of digital screens may decrease blink frequency, leading to an increased evaporation rate and an upsurge in the incidence and severity of DES. This study aims to assess the severity of DES symptoms and the risk factors among university students. A cross-sectional study was conducted at Umm AlQura University to evaluate the severity of DES among students and explore its potential association with digital screen use. Validated questionnaires were used to assess the severity of DES and digital screen usage. The study included 457 participants, of which 13% had symptoms suggestive of severe DES. Furthermore, multiple risk factors had a significant association with the severity of DES, including gender, use of monitor filters, monitor and room brightness, and smoking habits. DES symptoms were prevalent among university students, particularly female students. Although there was no significant association with the duration of screen usage and collage distribution. Other factors however, such as the usage of screen monitors and the brightness of both the monitor and the room, were significantly associated with the severity of DES symptoms.

## Introduction

Dry eye syndrome (DES) or dry eye disease (DED) is a tear film disorder caused by increased tear evaporation or decreased tear production^1^. DES is common in adults worldwide, affecting between 1 out of 5 to 1 out 11 individuals^2,3^. A study conducted among adults in Saudi Arabia, with the majority of participants being females, revealed that 38.4% of participants were diagnosed with DES. While another 36.8% experienced the same symptoms going undiagnosed. The high prevalence was attributed to environmental factors such as dry and hot weather in Saudi Arabia^4^.

A shift in learning methods characterizes the modern era; university students are extensively engaged with digital screens during online learning, leading to a substantial workload on their eyes. Excess use of digital screens, also called visual display terminals (VDT) can reduce blink frequency and facilitate tear evaporation, resulting in the development of DES^5^. Therefore, digital screening practices using laptops, tablets, television, and/or smartphones, should be investigated among university students, who are considered a high-risk group^6,7^.

Risk factors for DES include age, female gender, environmental factors such as air pollution, and digital screens^1,3^, have been identified in many previous studies. Although many studies have explored DES among university students, they yielded inconsistent results due to variations in geographical locations and methodologies. Therefore, we aim to assess the prevalence of DES symptoms among university students in our region and assess how different risk factors, mainly digital screen practices, can influence the severity of DES.

## Methods

### Study design

A cross-sectional study was conducted at Umm Alqura University (UQU) in Makkah, Saudi Arabia. Ethical approval was obtained from the Medical and Biological Ethics Research Committee of Umm Alqura University (No. HAPO-02-K-012-2023-12-1932), and all methods were performed in compliance with the relevant guidelines and regulations. We distributed an online questionnaire from May to June of 2023 among undergraduate students through the official university E-mail to four scientific and literary colleges using snowball sampling. Participants were informed that participation was voluntary. Visiting students from outside the university were excluded from the study.

### Questionnaire methods

Informed consent was obtained from all participants before starting the questionnaire. A specific question to verify UQU enrolment was used for inclusion criteria. Demographic data such as age, gender, and college affiliation were included in the questionnaire.

The severity of dry eye syndrome was assessed using the Ocular Surface Disease Index (OSDI), a twelve-question questionnaire categorized into eye symptoms, vision function, and environmental factors. Responses were rated on a scale from 0 (none of the time) to 4 (all the time) over the previous week. The sum score from the answered question was multiplied by twenty-five, divided by the number of all questions answered. Accordingly, the final score ranged from 0 to 100, categorized as the following: 0–13 normal, 13–22 mild, 23–32 moderate, and > 32 severe^8,9^.

A validated questionnaire was used to investigate digital screen habits^10^, which incorporated eight questions related to screen usage. Participants were asked about potential risk factors related to dry eye, including the duration of using glasses and contact lenses, smoking habits, and virtual reality device use. Participants were also asked about using artificial tears and dark mode on electronic devices and if they considered consulting an ophthalmologist for their symptoms.

### Sample size

To calculate the required sample size, we used OpenEpi version 3.0. The confidence interval is 95%, the frequency is 50%, and the design effect is 1. The total number of students at Umm Al-Qura University, based on the UQU official website, is 92,634^11^. The required sample size for this study is 383 participants. The number of participants based on the inclusion and exclusion criteria was 457.

### Statistical analysis

Microsoft Excel and Statistical Package for Social Studies (SPSS 26) were used for the analysis. Frequencies were calculated for categorical variables. The Chi-square test compared categorical variables. The numerical data was normally distributed. Therefore, mean and standard deviation were used. Univariate analysis was done to find the association between the severity of dry eye syndrome and gender, college, usage of screens, and risk factors. A p-value < 0.05 was considered significant.

## Results

**Figure 1:**
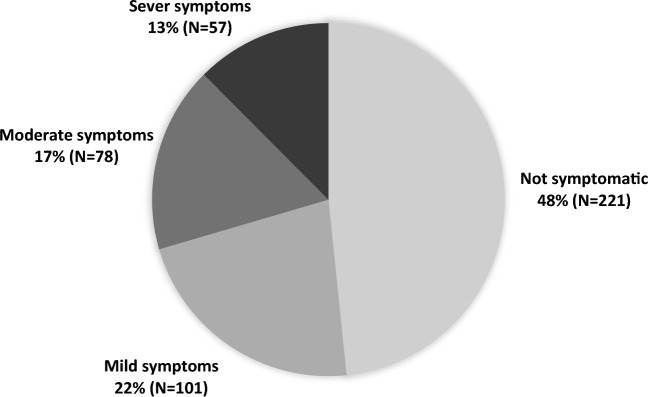
Prevalence and severity of dry eye syndrome.

The study includes 457 participants of both genders and various UQU colleges. The mean age was 22 years and two months, with a standard deviation of 2 years (Table 1). 48% (N = 221) of the participants did not complain of DES symptoms, whereas 52% (N = 236) experienced DES symptoms of varying severity (Fig. 1). Of note, 40.3% of participants reported their symptoms to a physician.

**Table 1:** Socio-demographic characteristics of study participants (N = 457).

Characteristic		Frequency	Percentage
Gender	Male	229	50.1%
	Female	228	49.9%
College	Medicine Colleges	278	60.8%
	Science and engineering colleges	70	15.3%
	Shari'ah and administration colleges	59	12.9%
	Colleges of humanities and educational sciences	50	10.9%
Age (mean ± SD)		22.2 ± 2	

*SD* Standard deviation.

Tables 2 and 3 highlight factors associated with the severity of DES. Gender exhibited a significant association (p-value =  < 0.001), with 60% of female participants exhibiting DES symptoms compared to 40% of males. Similarly, females tended to experience more severe DES symptoms than their male counterparts. Neither college affiliation demonstrated a significant difference in terms of DES prevalence nor screen usage factors including screen time duration, distance from the screen, seating position, screening breaking times and duration, awareness of the 20–20-20 rule, and usage of dark mode. However, other factors, such as the usage of monitor filters (p-value ≤ 0.001), screen brightness (p-value ≤ 0.001), and room brightness (p-value = 0.004), exhibited significant associations with DES symptom severity.

**Table 2:** Association of dry eye syndrome severity with socio-demographic data.

Demographic characteristics	The severity of dry eye syndrome				P-value
	Not symptomatic	Mild symptoms	Moderate symptoms	Severe symptoms	
Gender					
Male	134 (60.6%)	43 (42.6%)	34 (43.6%)	17 (29.8%)	< 0.001*
Female	87 (39.4%)	58 (57.4%)	44 (56.4%)	40 (70.2%)	
College					
Medicine colleges	142 (64.3%)	61 (60.4%)	42 (53.8%)	33 (57.9%)	0.407
Colleges of Humanities and Educational Sciences	20 (9%)	14 (13.9%)	8 (10.3%)	8 (14%)	
Science and engineering colleges	34 (15.4%)	12 (11.9%)	18 (23.1%)	6 (10.5%)	
Shariah and administration	25 (11.3%)	14 (13.9%)	25 (11.3%)	10 (17.5%)	

*Represent statistical significance data as a p-value of < 0.05.

**Table 3:** Association of dry eye syndrome severity with the usage of screens.

Demographic characteristics	The severity of ocular distress symptoms				P-value
	Not symptomatic	Mild symptoms	Moderate symptoms	Severe symptoms	
Duration of screen time usage per day					
< 2 h	2 (0.9%)	1 (1%)	2 (2.6%)	2 (3.5%)	0.056
2–4 h	47 (21.3%)	10 (9.9%)	15 (19.2%)	16 (28.1%)	
5 h or more	172 (77.8%)	90 (89.1%)	61 (78.2%)	39 (68.4%)	
Taking regular breaks from using screens					
Yes	158 (71.5%)	65 (64.4%)	51 (65.4%)	35 (61.4%)	0.363
No	63 (28.5%)	36 (35.6%)	27 (34.6%)	22 (38.6%)	
Regularity of breaks					
Every 30 min	47 (29.9%)	24 (36.4%)	11 (18.3%)	13 (31.7%)	0.301
Every 1 h	52 (33.1%)	24 (36.4%)	28 (46.7%)	14 (34.7%)	
> 1 h	58 (36.9%)	18 (27.3%)	21 (35%)	14 (34.7%)	
The average duration of breaks					
< 5 min	18 (11.4%)	8 (11.8%)	9 (14.5%)	7 (17.1%)	0.225
5–10 min	52 (32.9%)	20 (29.4%)	22 (35.5%)	15 (36.6%)	
11–15 min	28 (17.7%)	23 (33.8%)	16 (25.8%)	8 (19.5%)	
> 15 min	60 (38%)	17 (25%)	15 (24.2%)	11 (26.8%)	
Approximate distance between the eye and the screen					
< 40 cm	131 (59.3%)	67 (66.3%)	41 (52.6%)	28 (49.1%)	0.316
40–76 cm	75 (33.9%)	31 (30.7%)	32 (41%)	24 (42.1%)	
> 76 cm	15 (6.8%)	3 (3%)	5 (6.4%)	5 (8.8%)	
Seating position while using electronic devices					
Upright with a straight back	35 (15.8%)	16 (15.8%)	18 (23.1%)	13 (22.8%)	0.373
Lying back	101 (45.7%)	47 (46.5%)	26 (33.3%)	20 (35.1%)	
Bending back	85 (38.5%)	38 (37.6%)	34 (43.6%)	24 (42.1%)	
Usage of monitor filters					
Yes	47 (21.3%)	20 (19.8%)	21 (26.9%)	29 (50.9%)	< 0.001*
No	174 (78.8%)	81 (80.2%)	57 (74.1%)	28 (49.1%)	
Brightness of monitor					
Very bright	9 (4.1%)	4 (4%)	9 (11.5%)	12 (21.1%)	< 0.001*
Bright	68 (30.8%)	34 (33.7%)	29 (37.2%)	22 (38.6%)	
Dull	125 (56.6%)	57 (56.4%)	37 (47.4%)	19 (8.6%)	
Very dull	19 (8.6%)	6 (5.9%)	3 (3.8%)	4 (7%)	
Brightness of the room					
Very bright	11 (5%)	4 (4%)	4 (5.1%)	9 (15.8%)	0.004*
Bright	114 (51.6%)	59 (58.4%)	36 (46.2%)	25 (43.9%)	
Dull	82 (37.1%)	33 (32.7%)	30 (38.5%)	13 (22.8%)	
Very dull	14 (6.3%)	5 (5%)	8 (10.3%)	10 (17.5%)	
Participants aware of the 20–20-20 rule§					
Yes	79 (35.7%)	38 (37.6%)	33 (42.3%)	27 (47.4%)	0.377
No	142 (64.3%)	63 (62.4%)	45 (57.7%)	30 (52.6%)	
Usage of dark mode					
Yes	79 (35.7%)	38 (37.6%)	33 (42.3%)	27 (47.7%)	0.931
No	142 (64.3%)	63 (62.4%)	45 (57.7%)	30 (52.6%)	

*Represent statistical significance data as a p-value of < 0.05.

^§^The 20–20-20 rule stands for taking breaks: every 20 min, look at an object at least 20 feet away, for at least 20 s.

DES risk factors association with symptoms severity was evaluated and showed the following: usage of synthetic tears (p-value ≤ 0.001), wearing glasses (p-value ≤ 0.001), wearing contact lenses (p-value ≤ 0.001), smoking habits (p-value ≤ 0.001), and usage of virtual reality (VR) glasses (p-value ≤ 0.001), wearing glasses (p-value = 0.003) (Table 4).

**Table 4:** Association of dry eye syndrome severity with multiple risk factors of dry eye syndrome.

Demographic characteristics	The severity of dry eye syndrome				P-value
	Not symptomatic	Mild symptoms	Moderate symptoms	Severe symptoms	
Frequency of using synthetic tears					
Daily	5 (2.3%)	2 (2%)	14 (17.9%)	5 (8.8%)	< 0.001*
Multiple times a week	15 (6.8%)	20 (19.8%)	14 (17.8%)	14 (24.6%)	
Rarely	65 (29.4%)	28 (27.7%)	24 (30.8%)	29 (50.9%)	
Do not use it	136 (61.5%)	51 (50.5%)	26 (33.3%)	9 (15.8%)	
Duration of wearing glasses					
< 1 year	9 (4.1%)	8 (7.9%)	10 (12.8%)	8 (14%)	0.003*
1–5 years	32 (14.5%)	17 (16.8%)	22 (28.2%)	9 (15.8%)	
6–10 years	26 (11.8%)	10 (9.9%)	10 (12.8%)	11 (19.3%)	
> 10 years	16 (7.2%)	8 (7.9%)	7 (9%)	7 (12.3%)	
Do not use glasses	138 (62.4%)	58 (57.4%)	22 (28.2%)	9 (15.8%)	
Regularity of wearing contact lenses					
Daily (> 6 h)	2 (0.9%)	1 (1%)	5 (6.4%)	2 (3.5%)	< 0.001*
Daily (< 6 h)	1 (0.5%)	0 (0%)	0 (0%)	4 (7%)	
Multiple times a week	14 (6.3%)	6 (5.9%)	11 (14.1%)	15 (26.3%)	
Rarely	34 (15.4%)	28 (27.7%)	24 (30.8%)	13 (22.8%)	
Do not use them	170 (76.9%)	66 (65.3%)	38 (48.7%)	23 (40.4%)	
Smoking habits					
> 30 cigarettes/day	2 (0.9%)	0 (0%)	1 (1.3%)	0 (0%)	< 0.001*
21–30 cigarettes/day	0 (0%)	0 (0%)	2 (2.6%)	5 (8.8%)	
11–20 cigarettes/day	13 (5.9%)	4 (4%)	7 (9%)	3 (5.3%)	
1–10 cigarettes/day	8 (3.6%)	4 (4%)	3 (3.8%)	5 (8.8%)	
Previous smoker	8 (3.6%)	7 (6.9%)	3 (3.8%)	5 (8.8%)	
Do not smoke	190 (86%)	86 (85.1%)	62 (79.5%)	39 (68.4%)	
Using Virtual Reality (VR) glasses					
Daily (> 6 h)	0 (0%)	0 (0%)	0 (0%)	1 (1.8%)	< 0.001*
Daily (< 6 h)	0 (0%)	0 (0%)	1 (1.3%)	2 (3.5%)	
Multiple times a week	2 (0.9%)	1 (1%)	3 (3.8%)	7 (12.3%)	
Rarely	15 (6.8%)	10 (9.9%)	13 (16.7%)	8 (14%)	
Do not use them	204 (92.3%)	90 (89.1%)	61 (78.2%)	39 (68.4%)	

*Represent statistical significance data as a p-value of < 0.05.

## Discussion

While several studies have explored DES symptoms among university students worldwide, there is a scarcity of research evaluating DES in Saudi Arabian universities and exploring its correlation with risk factors. This study found that more than half of the participants experienced dry eye symptoms with varying degrees of severity. In comparison, researchers in Thailand found that less than 1 out of 10 students have dry eye disease^12^. This considerable disparity in the prevalence of DES symptoms could be related to the influence of the local environment and climate. Thailand's tropical high-humidity climate differs significantly from the Middle East desert climate, and this divergence in climate and humidity may play a crucial role in maintaining ocular moisture and reducing tear evaporation. This conclusion is supported by a study conducted by Berg et al., indicating a strong correlation between humidity levels and the eye's dryness^13^.

The study result indicates a significant variation in the severity of DES based on gender, as females show more severe DES than males. This finding correlates with the results of three cross-sectional studies conducted in Al-Ahsa, Saudi Arabia^14^, The United Arab Emirates^15^, and the United States^16^, which demonstrate that female has more severe DES than males. Although the exact reason for this gender-related variation is not clear, it has been suggested that hormonal changes in females may contribute to this finding^17^.

Surprisingly, we did not find a significant correlation between the severity of DES and the duration of screen time. In contrast, several studies found a strong relationship between DES severity and digital screen use^18^. Another Saudi study has a similar result of increasing DES severity and long duration of using digital screens^4^. The discrepancy in results between this study and others may be attributed to students' young age or other confounders, for example, the use of protective tools from screen light like blue light filters. Blue light has been implicated in exacerbating dry eye symptoms by causing oxidative damage to the epithelium cells of the cornea and leading to the worsening of dry eye symptoms^19^.

In today's world, the daily use of digital devices and prolonged exposure to digital screen lights are inevitable. However, there are modifiable factors that can help reduce the prevalence and severity of DES. Screen brightness is one of the most important modifiable factors, as this study found a strong correlation between screen brightness and the severity of DES symptoms. This finding is substantiated by several published studies. For example, a cross-sectional study conducted in India suggests reducing the screen's brightness can be an effective method to lower the risk of developing dry eye^20^. Decreasing the screen's brightness will reduce the amount of light exposure to the eyes, which may decrease eye strain and tear evaporation. In addition, decreasing eye strain promotes regular frequency of eye blinking^21^.

The study's limitation is it did not include clinical assessments, such as visual acuity and the Schrimmer test. A notable strength of our study lies in using a validated instrument (OSDI) to assess the severity of DES. However, future research, including clinical assessment of eye dryness, is highly recommended to enhance diagnostic accuracy.

## Conclusion

This study revealed a high prevalence of DES symptoms among university students, particularly female students, in Makkah, Saudi Arabia. Although there was no significant association with the duration of screen usage and collage distribution. Other factors however, such as the usage of screen monitors and the brightness of both the monitor and the room, were significantly associated with the severity of DES symptoms.

## Data Availability

All the data related to this study are available from the corresponding author upon reasonable request.
